# CpG immunostimulatory oligodeoxynucleotide 1826 as a novel nasal ODN adjuvant enhanced the protective efficacy of the periodontitis gene vaccine in a periodontitis model in SD rats

**DOI:** 10.1186/s12903-021-01763-1

**Published:** 2021-08-16

**Authors:** Guohui Bai, Hang Yu, Xiaoyan Guan, Fengjiao Zeng, Xia Liu, Bin Chen, Jianguo Liu, Yuan Tian

**Affiliations:** 1 Key Laboratory of Oral Disease Research, School of Stomatology, Zunyi Medical University, Zunyi, 563000 China; 2 Hospital of Stomatology, Zunyi Medical University, Zunyi, 563000 China

**Keywords:** Periodontitis, Periodontitis gene vaccine, CpG-ODN, Alveolar bone loss, COX-2, RANKL

## Abstract

**Background:**

We previously demonstrated that nasal administration of periodontitis gene vaccine (pVAX1*-HA2-fimA*) or pVAX1*-HA2-fimA* plus IL-15 as adjuvant provoked protective immunity in the periodontal tissue of SD rats. This study evaluated the immune effect of pVAX1*-HA2-fimA* plus CpG-ODN 1826 as an adjuvant in the SD rat periodontitis models to improve the efficacy of the previously used vaccine.

**Methods:**

Periodontitis was induced in maxillary second molars in SD rats receiving a ligature and infected with *Porphyromonas gingivalis*. Forty-two SD rats were randomly assigned to six groups: A, control without *P. gingivalis*; B, *P. gingivalis* with saline; C, *P. gingivalis* with pVAX1; D, *P. gingivalis* with pVAX1*-HA2-fimA*; E, *P. gingivalis* with pVAX1*-HA2-fimA/IL-15*; F, *P. gingivalis* with pVAX1*-HA2-fimA*+CpG ODN 1826 (30 µg). The levels of FimA-specific and HA2-specific secretory IgA antibodies in the saliva of rats were measured by ELISA. The levels of COX-2 and RANKL were detected by immunohistochemical assay. Morphometric analysis was used to evaluate alveolar bone loss. Major organs were observed by HE staining.

**Results:**

30 μg could be the optimal immunization dose for CpG-ODN 1826 and the levels of SIgA antibody were consistently higher in the pVAX1*-HA2-fimA*+CpG-ODN 1826 (30 µg) group than in the other groups during weeks 1–8 (*P* < 0.05, except week 1 or 2). Morphometric analysis demonstrated that pVAX1*-HA2-fimA*+CpG-ODN 1826 (30 µg) significantly reduced alveolar bone loss in ligated maxillary molars in group F compared with groups B–E (*P* < 0.05). Immunohistochemical assays revealed that the levels of COX-2 and RANKL were significantly lower in group F compared with groups B–E (*P* < 0.05). HE staining results of the major organs indicated that pVAX1*-HA2-fimA* with or without CpG-ODN 1826 was not toxic for in vivo use.

**Conclusions:**

These results indicated that CpG-ODN 1826 (30 µg) could be used as an effective and safe mucosal adjuvant for pVAX1*-HA2-fimA* in SD rats since it could elicit mucosal SIgA responses and modulate COX-2 and RANKL production during weeks 1–8, thereby inhibiting inflammation and decreasing bone loss.

## Background

Periodontitis is one of the most common oral diseases worldwide and leads to the destruction of periodontal tissue, mainly in the form of inflammation of the gingiva and alveolar bone loss [1]. Besides, periodontitis is a risk factor for various systemic diseases, including cardiovascular disease, diabetes, and osteoporosis [2–4]. The Gram-negative anaerobic bacterium *Porphyromonas gingivalis* (*P. gingivalis*) is one of the key pathogens in chronic periodontitis [5]. Multiple studies have demonstrated that *P. gingivalis* could produce many virulent factors, such as gingipains, lipopolysaccharide (LPS), and fimbriae/pili, to destruct the periodontal tissue on their own or act through other mediators to induce inflammation [6, 7]. For instance, it is thought that *P. gingivalis* needed exogenous porphyrin and iron to survive [8]. In the inflamed periodontal pocket, cysteine proteases bound heme with domains such as hemagglutinin-2 (HA2), and high-affinity HA2-hemin binding would be a ready source of heme-associated porphyrin and iron required for growth and virulence and participate in cell-surface heme deposition [9, 10]. The polymer of the fimA protein (fimbrillin), encoded by the gene fimA, is an important virulence factor of *P. gingivalis* [11–13]. It plays an important role in the pathogenesis of *P. gingivalis*, which is closely related to periodontitis [14].

In previous studies, we developed a gene vaccine (pVAX1*-HA2-fimA*) for periodontitis. Those studies have demonstrated that nasal administration of pVAX1*-HA2-fimA* induced FimA-specific and HA2-specific secretory immunoglobulin A (S-IgA) antibodies in saliva, thereby reducing alveolar bone loss due to oral infection with *P. gingivalis* [15–18]. It is well known that one of the main tasks in the development of periodontitis vaccines is to improve their immunogenicity. Traditionally, vaccines, especially mucosal vaccines, generally require the use of adjuvants to achieve these objectives. This study aimed to investigate whether the immunogenicity of the periodontitis gene vaccine (pVAX1*-HA2-fimA*) could be further improved by including some adjuvants.

In recent years, CpG oligodeoxynucleotides (CpG-ODNs) have been widely used as new options for vaccine adjuvants based on their strong capacity to safely enhance vaccine immunogenicity. The unmethylated CpG-ODNs are ligands and agonists for human and mouse toll-like receptor 9 (TLR9). When CpG-ODNs served as vaccine adjuvants, they could enhance the induction of vaccine-specific humoral and cellular immune responses [19–24]. For example, Kataoka et al*.* showed that nasal CpG-ODN effectively enhanced dendritic cells and supplied balanced rFimA-specific IgA protective immunity against *P. gingivalis* in the respiratory tract [19]. Khodadadi et al. demonstrated that combined use of CpG-ODN adjuvant enhanced the immune protection and efficacy of a multi-epitope DNA vaccine [20]. So far, three major types of immunostimulatory CpG ODNs (A, B, or C type) have been well characterized. B-type CpG ODNs contain one or more CpG motifs with phosphorothiolate bond instead of phosphodiester linkage. The phosphorothiolate bond enhances resistance to DNase digestion and substantially prolongs in vivo half-life [25]. B-type CpG ODNs could markedly activate B cells to produce antibodies, but induce relatively little IFN-α secretion or natural killer (NK) cell activity [26]. CpG-ODN 1826, a well-known B-type CpG, has been successfully tested in various vaccination models and exerted an immune enhancing effect on its own or when used as an adjuvant for vaccines [27–29].

In this study, to study the influence of CpG-ODNs on the immunogenicity of periodontitis gene vaccine (pVAX1*-HA2-fimA*), a vaccine containing CpG-ODN 1826 (5’-TCC ATG ACG TTC CTG ACG TT-3’) was assembled. We evaluated the specific immune responses and protective efficacy in SD rats given a periodontitis gene vaccine (pVAX1*-HA2-fimA*) plus CpG-ODN 1826 adjuvant to prevent oral infection by *P. gingivalis* by measuring the levels of SIgA antibody, alveolar bone loss, and immune-inflammatory modulation. Besides, we also compared the effect of CpG-ODN 1826 with that of the previously used IL-15 adjuvant to our pVAX1-*HA2-fimA* periodontitis gene vaccine in this study.

## Methods

### Animals

Forty-two, 4- to 6-week-old, healthy, male Sprague Dawley (SD) rats (100 ± 10 g) were included in the study. The animals were purchased from TianQin Biotechnology Company of Changsha and kept in the Special Key Laboratory of Oral Diseases, Higher Education Institution in Guizhou Province. This study was approved by the medical ethics committee of Zunyi Medical University (Approval No. YJSKTLS-2018-2021-034A) and all studies involving animals were reported in accordance with the ARRIVE guidelines for reporting experiments involving animals [30].

### Antigen and adjuvant

Plasmid pVAX1 expressing pVAX1-*HA2-fimA* and pVAX1-*HA2-fimA/IL-15* was constructed by the research group in the early stage and stored in the Special Key Laboratory of Oral Diseases, Higher Education Institution in Guizhou Province, as described previously [31]. The purity of pVAX1-*HA2-fimA* and pVAX1-*HA2-fimA/IL-15* was determined by SDS-PAGE, and no contaminating protein bands were noted.

### Experimental groups

First, six groups of SD rats (3 SD rats/group) were administered drops to bilateral nasal mucosa to detect the expression of FimA-specific and HA2-specific secretory IgA antibodies in the saliva of SD rats. Control groups were administered to bilateral nasal mucosa with 100 ug of pVAX1 (group I), 100 ug of pVAX1-*HA2-fimA* (group II), 100 ug of pVAX1-*HA2-fimA/IL-15* (group III), respectively. Experimental groups were administered to bilateral nasal mucosa with 100 ug of pVAX1-*HA2-fimA*+CpG-ODN 1826 (10 μg) (group IV), 100 ug of pVAX1-*HA2-fimA*+CpG-ODN 1826 (30 μg) (group V), and 100 ug of pVAX1-*HA2-fimA*+CpG-ODN 1826 (50 μg) (group VI), respectively. Each SD rat was administered drops to bilateral nasal mucosa on weeks 0, 1, and 3. Before immunization by nasal mucosal drip and during the 1st-10th weeks after immunization, rats were injected weekly with 2% trichothecene drops (0.2 mg/100 g body weight) subcutaneously through the neck, and 0.5 mL of saliva was collected after 5 min of injection, followed by centrifugation at 4 °C for 10 min (12,000 rpm), and impurities were discarded for detection using the ELASA method as described elsewhere [32].

Next, Forty-two SD rats were randomly assigned to the blank group (group A), saline group (group B), pVAX1 group (group C), pVAX1-*HA2-fimA* group (group D), pVAX1-*HA2-fimA/IL-15* group (group E), and pVAX1-*HA2-fimA*+CpG-ODN 1826 (30 µg) group (group F) with 7 SD rats in each group. Except for group A, the remaining 5 groups of 35 SD rats were used to construct periodontitis models. Subsequently, the SD rats in the group B–F were administered to bilateral nasal mucosa with 100 ug of saline, 100 ug of pVAX1, 100 ug of pVAX1-*HA2-fimA*, 100 ug of pVAX1-*HA2-fimA/IL-15*, and 100 ug of pVAX1-*HA2-fimA*+CpG-ODN 1826 (30 μg) on weeks 0, 1 and 3, respectively. Before immunization by nasal mucosal drip and during the 1st–5th weeks after the last immunization (on week 3), the saliva of 42 SD rats was collected using the same method described above, and the levels of FimA-specific and HA2-specific secretory IgA antibodies were measured separately using the ELISA method.

### Establishment of SD rat periodontitis models

Seven SD rats of group A and 35 immunized SD rats of group B–F were used to establish periodontitis models by the following surgery. At week 4, rats in all groups (A–F) were anesthetized with 10% chloral hydrate (0.3 mL/100 g). A 4–0 wire was placed in the dentogingival area of both maxillary second molars. Rats in the experimental groups (B–F) were inoculated with 200 µL (1 × 10^9^ CFU/mL) of *P. gingivalis* at the silk ligation, and the control group (A) was inoculated with 200 µL of BHI broth. Inoculation was performed three times a day for three consecutive days. The gingival status, tooth looseness, and wire loss were observed weekly, and ligation was performed again after anesthesia if there was wire loss. One rat from each of group A and group C was executed by excessive anesthesia at week 8, and the maxilla was dissected and separated. Histopathological sections of the maxilla on one side were observed by HE staining using the method as described elsewhere [33], and the maxilla on the other side was treated to observe the degree of alveolar bone resorption under a microscope and photographed.

### Histopathological examination

All SD rats in group A–F were executed at week 8 to obtain organs and maxillary periodontal tissues. SD rat organs, including heart, liver, spleen, lung, and kidney, were harvested and preserved in 10% buffered formalin, followed by HE staining treatment. All maxillary periodontal tissues were rinsed in running tap water for 12 h. Next, the samples were fixed in 4% paraformaldehyde at 4 °C for 24 h and placed in 10% ethylenediaminetetraacetic acid (EDTA) for decalcification for 30 days. Subsequently, samples were rinsed in phosphate-buffered saline (PBS), dehydrated, embedded, and cut into 5 μm sections using a Leica microtome (Leica, Germany). After antigen retrieval and blocking were performed, the tissue sections were incubated with 3% H_2_O_2_ in methanol for 10 min to quench endogenous peroxidase activity. The sections were then rinsed with phosphate-buffered saline (PBS) and blocked with 5% BSA for 1 h and incubated with primary antibodies against COX-2 (1:100, ab179800; Proteintech Group, Inc, Wuhan City, Hubei, China) and RANKL (1:100, ab182158; Proteintech Group, Inc, Wuhan City, Hubei, China) at 37 °C for 2 h. After being washed, sections were incubated with the secondary antibody (Goat Anti-Rat IgG) for 40 min at room temperature (1:500 to 1:2,000, Abcam, Cambridge, UK). After being rinsed with PBS, 50 μL of 3,3′-diaminobenzidine (DAB) solution was added, and the mixture was left to react for 5 min. The sections were stained with hematoxylin for 1 min and rinsed with water for 1 min. Integrated optical density quantified using Image-Pro Plus 5.0 software was used to assess the expression of COX-2 and RANKL. Three views from each sample were selected for mean value calculations, and group means were calculated using the mean of the individual measurements.

### Measurement of alveolar bone loss

After the sacrifice by excessive anesthesia, the rat maxillaries were boiled in deionized water for 15 min, the periodontal soft tissues were gently removed, and then soaked in 3% hydrogen peroxide for 24 h and stained with 1% methylene blue. A stereomicroscope (Leica, Switzerland) was used for observing the morphology of the alveolar bone of each group of rats. Image J software was used to measure the distance between the cemento-enamel junction and alveolar bone crest along the axis of the teeth. To assess the average alveolar bone loss, three points were measured along the axis of the teeth in the buccal and lingual regions. The average bone loss was calculated for each tooth.

### Statistical analysis

The results were expressed as means ± standard deviations. Statistical differences among groups were detected by one-way analysis of variance (ANOVA) using GraphPad Prism 7 (GraphPad Inc., La Jolla, CA, USA). A *P* value of < 0.05 was considered to be statistically significant.

## Results

### Periodontitis models establishment and evaluation

**Fig. 1:**
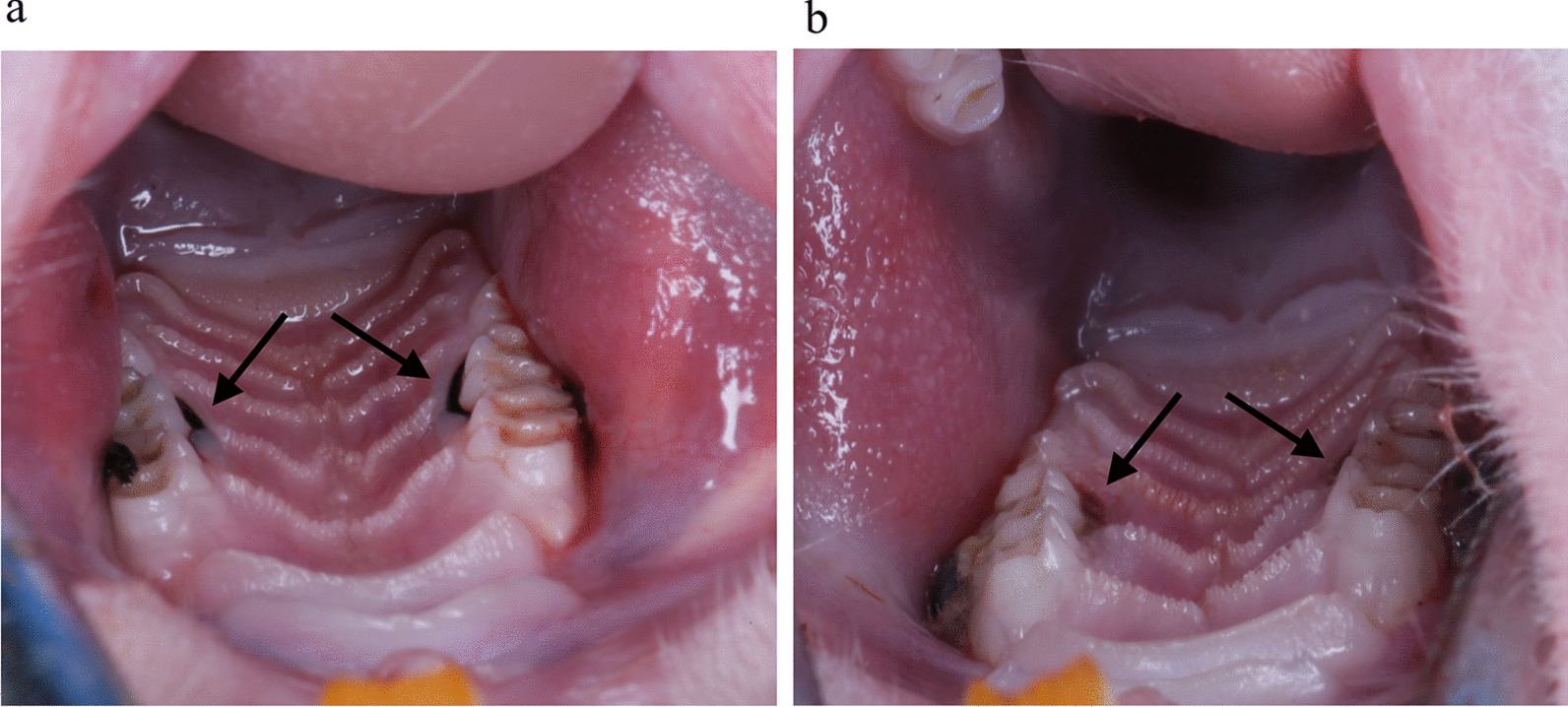
Establishment of periodontitis model in SD rats. **a** At week 4. **b** At week 8

**Fig. 2:**
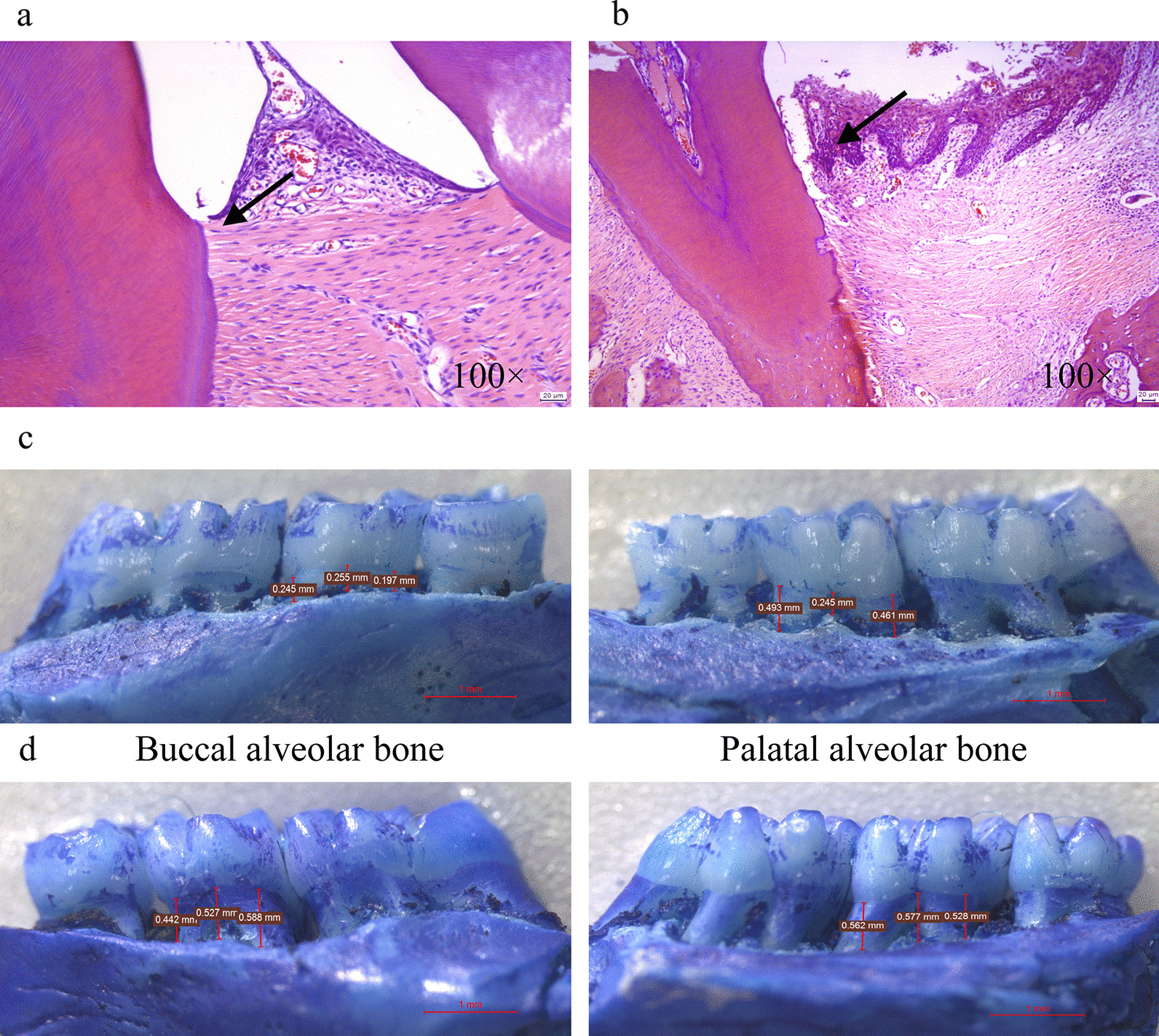
Evaluation of periodontitis model in rats at week 8. HE staining result of the maxilla on one side of one rat from each of blank group (**a**) and pVAX1 group (**b**). Scale bars represent 20 μm. Magnification, 100×. Detection of buccal alveolar bone and palatal alveolar bone damage of the maxilla on the other side of one rat from each of blank group (**c**) and pVAX1 group (**d**) by stereomicroscopy and immunohistochemical staining

After the periodontitis model was established at week 4, the periodontal condition of the maxillary second molars of SD rats in group C was examined, and it was found that there was no significant redness and swelling of gingiva, no loosening of teeth, and no obvious periodontal pocket formation (Fig. 1a). Gingival redness and swelling, tooth loosening, bleeding on probing and significant periodontal pocket formation in the maxillary second molars of the rats could be observed at week 8 (Fig. 1b). In addition, compared with the blank group (Fig. 2a), HE staining results showed that the gingival sulcus epithelium of the rats in the pVAX1 group showed erosion or ulceration, separation of the bound epithelium from the tooth surface, and proliferation of the epithelium toward the root, forming periodontal pockets. The gingival sulcus epithelium and the connective tissue beneath the bound gingival sulcus epithelium were degenerated and destroyed and formed structureless material (Fig. 2b). The alveolar bone height of rats in the pVAX1 group (Fig. 2d) was significantly reduced compared with the blank group (Fig. 2c) by stereomicroscopic examining. All the above results proved that the rat periodontitis model was successfully constructed.

### Periodontitis gene vaccine-induced salivary secretory IgA (sIgA) antibodies expression

**Fig. 3:**
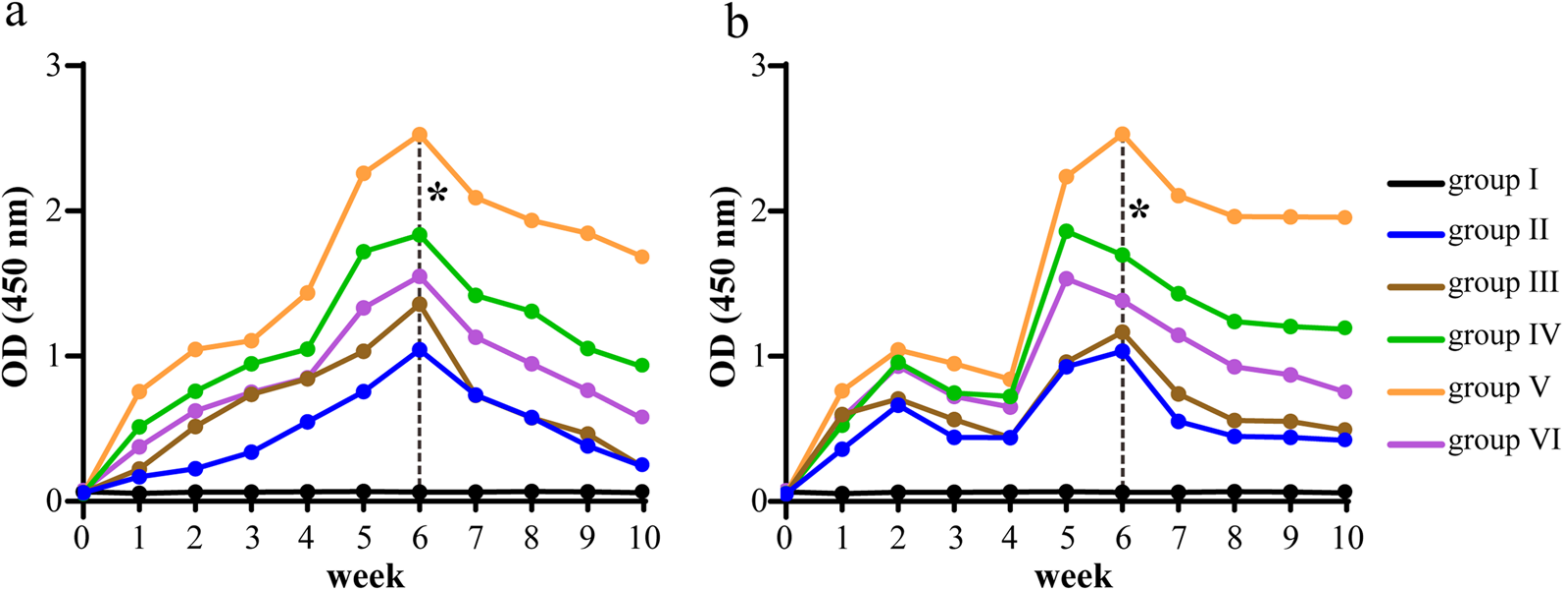
Expression levels of FimA-specific (**a**) and HA2-specific (**b**) secretory IgA antibodies in the saliva of SD rats at week 0–8, which were immunized with 100 ug of pVAX1 (group I), 100 ug of pVAX1-*HA2-fimA* (group II), 100 ug of pVAX1-*HA2-fimA/IL-15* (group III), 100 ug of pVAX1-*HA2-fimA*+CpG-ODN 1826 (10 μg) (group IV), 100 ug of pVAX1-*HA2-fimA*+CpG-ODN 1826 (30 μg) (group V) and 100 ug of pVAX1-*HA2-fimA*+CpG-ODN 1826 (50 μg) (group VI), respectively. Asterisk represents a significant difference between group V and the other groups, respectively, *P < 0.05

The levels of FimA-specific and HA2-specific secretory IgA antibodies in the saliva of rats in groups I-VI were measured by ELISA from week 0 to 10, and the results showed that the levels of SIgA antibody in all groups, except the blank group, started to increase from week 1, reached the highest level at week 5 or week 6, and then started to decrease (Fig. 3a, b). The SIgA antibody levels in the pVAX1-*HA2-fimA*+CpG-ODN 1826 (30 μg) group were consistently higher than those in the other groups, significantly higher at week 6 (*P* < 0.05), and remained at a high level at week 10. Therefore, 30 μg could be the optimal immunization dose for CpG-ODN 1826 and used in subsequent experiments (Fig. 3a, b).

**Fig. 4:**
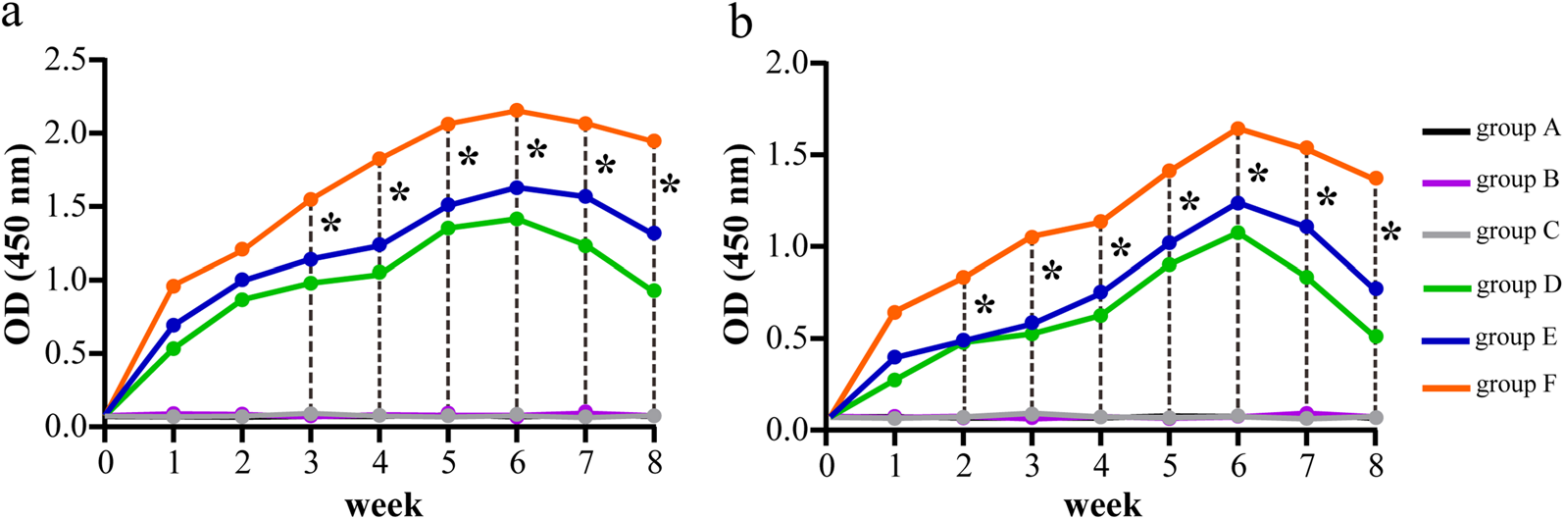
Expression levels of FimA-specific (**a**) and HA2-specific (**b**) secretory IgA antibodies in the saliva of SD rats at week 0–8, which were immunized with nothing (group A), 100 ug of saline (group B), 100 ug of pVAX1 (group C), 100 ug of pVAX1-*HA2-fimA* (group D), 100 ug of pVAX1-*HA2-fimA/IL-15* (group E), and 100 ug of pVAX1-*HA2-fimA*+CpG-ODN 1826 (30 μg) (group F), respectively. Asterisk represents a significant difference between group F and the other groups, respectively, *P < 0.05

Similarly, the levels of FimA-specific and HA2-specific secretory IgA antibodies in the saliva of rats in the groups A–F were measured by ELISA from weeks 1 to 8, respectively. The results showed that the antibody levels in all groups except the blank group (group A), the saline group (group B) and the pVAX1 group (group C) increased rapidly in the first week after immunization, reached the highest level at week 6, and then decreased slowly (Fig. 4a, b). The levels of SIgA antibody were consistently higher in the pVAX1-*HA2-fimA*+CpG-ODN 1826 group (30 µg) than in the other groups during weeks 1–8 (*P* < 0.05, except week 1 or 2) (Fig. 4a, b).

### Alveolar bone loss

**Fig. 5:**
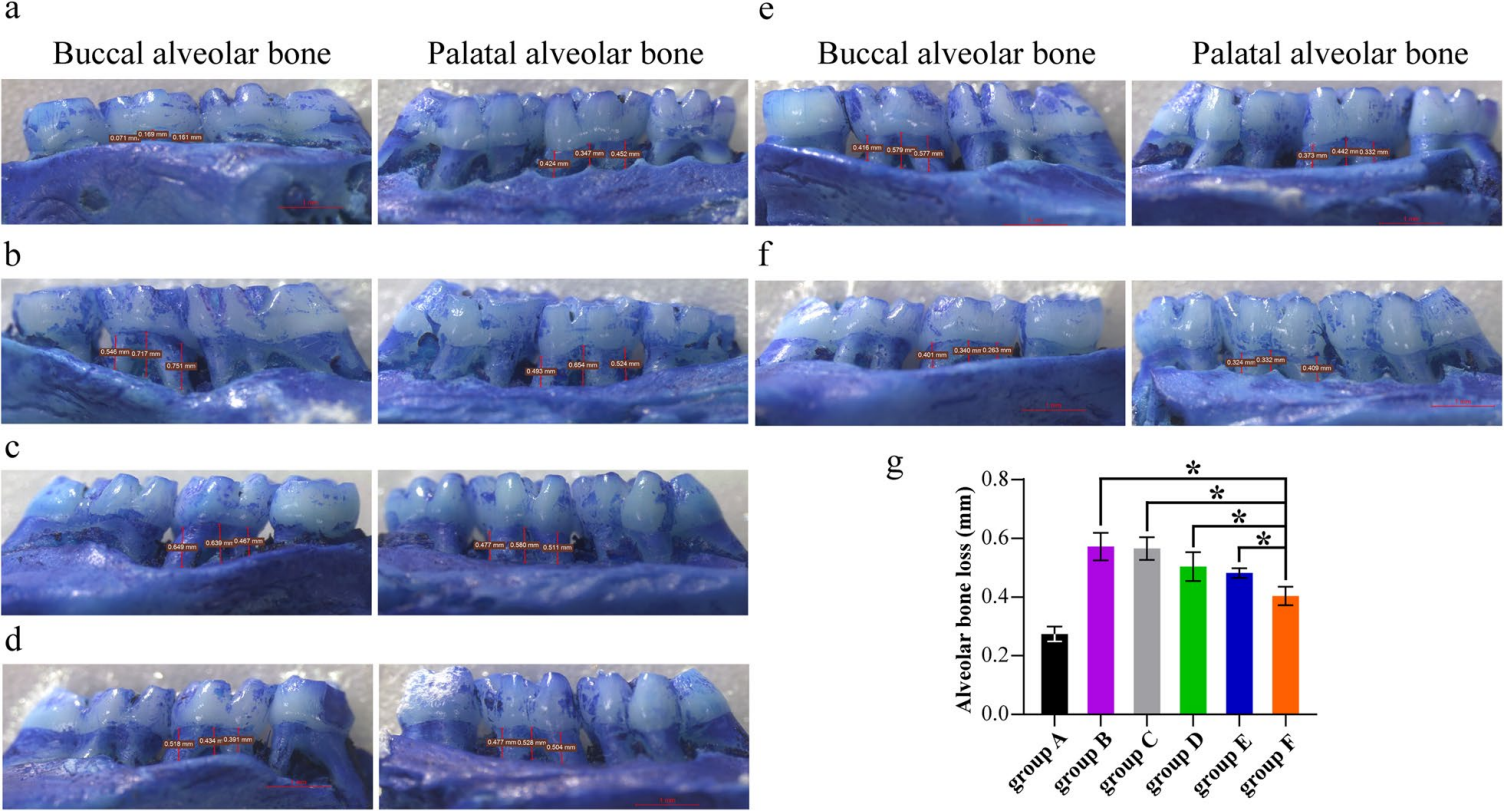
Detection of rat alveolar bone damage by stereomicroscopy and immunohistochemical staining. Buccal and palatal alveolar bone loss were measured along the long axis of the teeth in **a** group A (blank), **b** group B (100 ug of saline), **c** group C (100 ug of pVAX1), **d** group D (100 ug of pVAX1-*HA2-fimA*), **e** group E (100 ug of pVAX1-*HA2-fimA/IL-15*), and **f** group F (100 ug of pVAX1-*HA2-fimA*+CpG-ODN 1826 (30 μg)), respectively. **g** Statistical results. Asterisk represents a significant difference between group F and group B–E, respectively, *P < 0.05

A stereomicroscope observation showed that rats in groups B and C had severe alveolar bone destruction (Fig. 5b, c) compared with group A (Fig. 5a), and the administration of pVAX1-*HA2-fimA* with or without IL-15 or CpG-ODN 1826 inhibited the destruction of alveolar bone in the maxillary second molars of rats during the establishment of the rat periodontitis models (Fig. 5d–f). Severe alveolar bone destruction in rats in group B and C was characterized by an increased distance between the enamel-cement junction and the alveolar bone crest of the second molars (571.97 ± 47.07 μm and 562.88 ± 39.43 μm, respectively) (Fig. 5b, c). The mean distance between the enamel-cement junction and the alveolar bone crest (along the axis of the teeth) was 503.44 ± 49.46 μm and 481.77 ± 17.12 μm in the second maxillary molars in groups D and group E, respectively (Fig. 5d, e). pVAX1-*HA2-fimA*+CpG-ODN 1826 (30 µg) significantly reduced alveolar bone loss in ligated maxillary molars in group F compared with groups B–E (*P* < 0.05) (Fig. 5g).

### Expression of COX-2 and RANKL

**Fig. 6:**
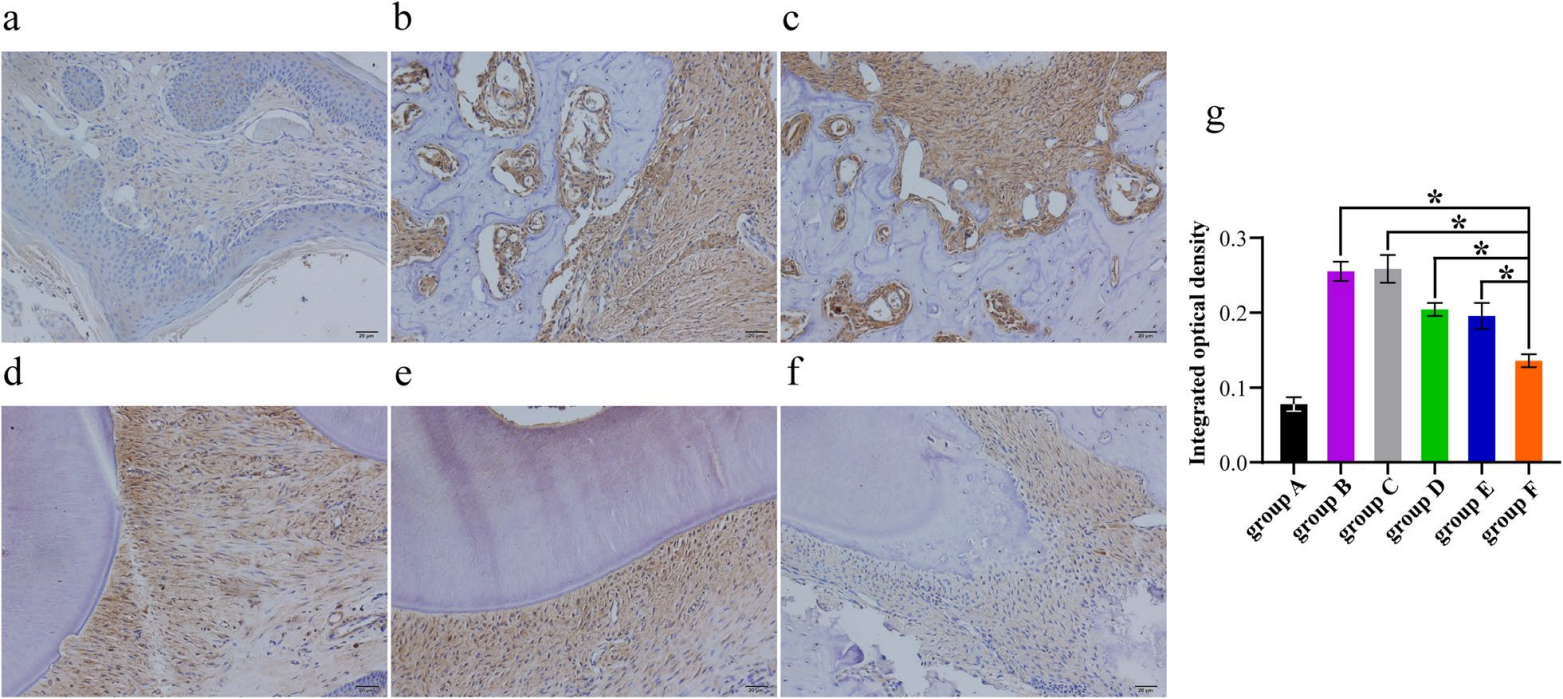
Expression of COX-2 in the periodontal tissue of the maxillary second molars of SD rats in group A-F. **a** group A (blank), **b** group B (100 ug of saline), **c** group C (100 ug of pVAX1), **d** group D (100 ug of pVAX1-*HA2-fimA*), **e** group E (100 ug of pVAX1-*HA2-fimA/IL-15*), and **f** group F (100 ug of pVAX1-*HA2-fimA*+CpG-ODN 1826 (30 μg)), **g** statistical results. Asterisk represents a significant difference between group F and group B–E, respectively, *P < 0.05. Scale bars represent 20 μm. Magnification, 200×

**Fig. 7:**
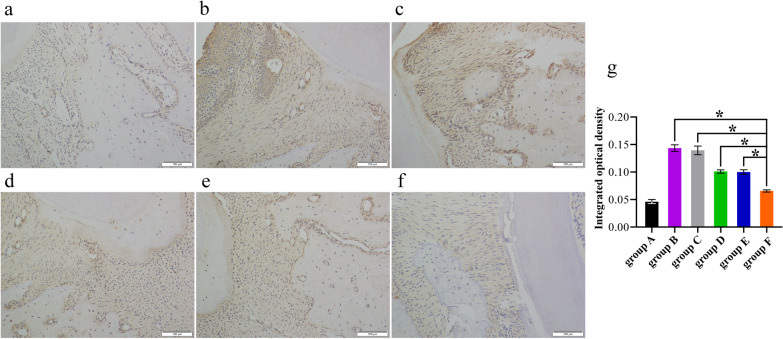
Expression of RANKL in the periodontal tissue of the maxillary second molars of SD rats in group A-F. **a** group A (blank), **b** group B (100 ug of saline), **c** group C (100 ug of pVAX1), **d** group D (100 ug of pVAX1-*HA2-fimA*), **e** group E (100 ug of pVAX1-*HA2-fimA/IL-15*), and **f** group F [100 ug of pVAX1-*HA2-fimA*+CpG-ODN 1826 (30 μg)], **g** Statistical results. Asterisk represents a significant difference between group F and group B–E, respectively, *P < 0.05. Scale bars represent 100 μm. Magnification, 200×

The result of immunohistochemical staining showed that there was a large amount of COX-2 and RANKL in the periodontal tissue of the maxillary second molars of SD rats in groups B–F compared with group A. Specifically, the integrated optical densities of COX-2 in the groups B–F were 0.255 ± 0.0128, 0.257 ± 0.0188, 0.204 ± 0.0088, 0.195 ± 0.0172, and 0.135 ± 0.0085, respectively (Fig. 6). The integrated optical densities of RANKL in the groups B–F were 0.144 ± 0.0061, 0.139 ± 0.0079, 0.101 ± 0.0028, 0.100 ± 0.0041, and 0.065 ± 0.0021, respectively (Fig. 7). The levels of COX-2 and RANKL were significantly lower in the periodontal tissue of the maxillary second molars of SD rats in group F compared with groups B–E (*P* < 0.05) (Figs. 6g, 7g).

### Histopathological study on organs of rat-immunized periodontitis gene vaccines

**Fig. 8:**
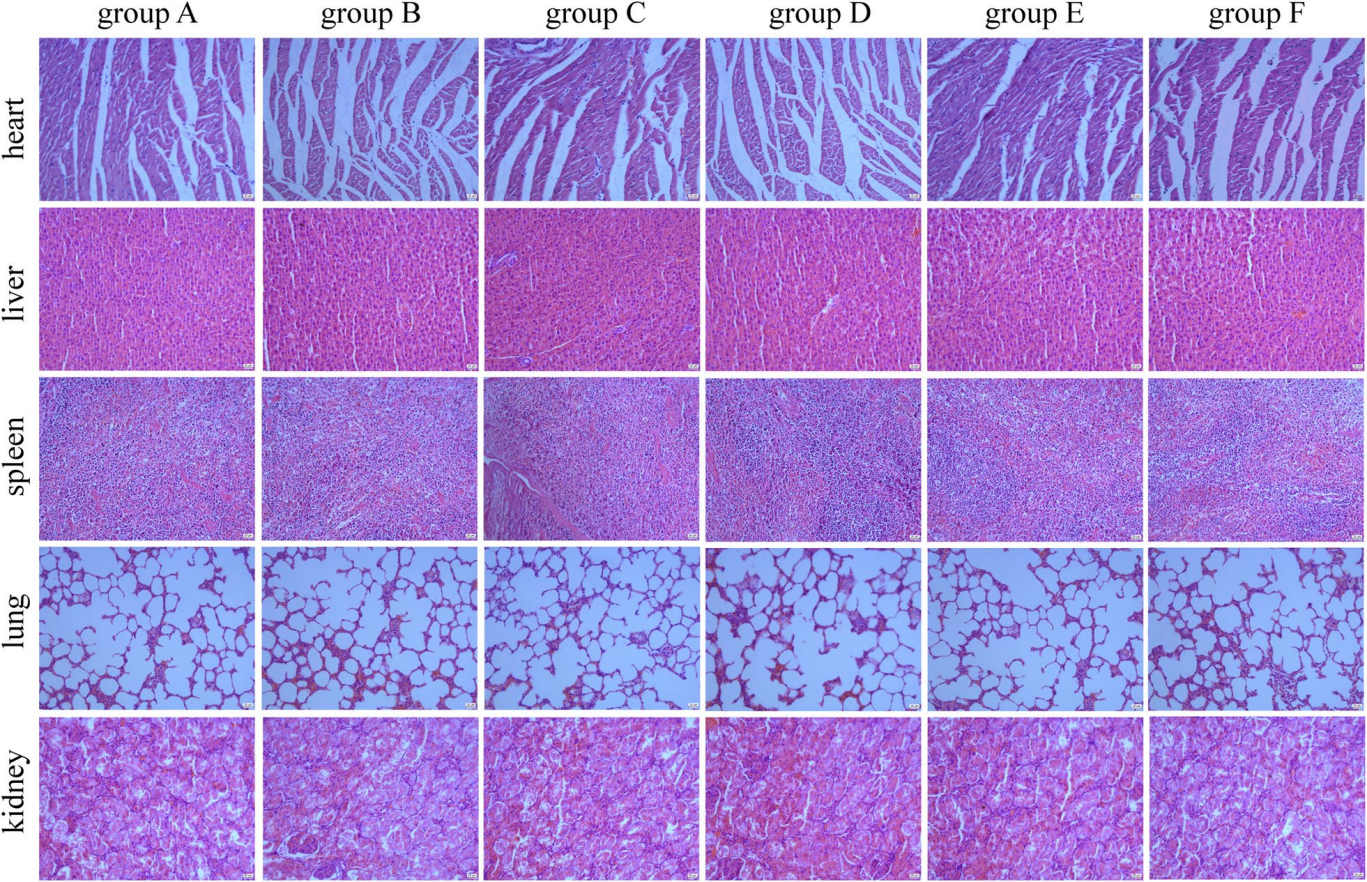
Histopathological examination of organs from SD rats, which were immunized with nothing (group A), 100 ug of saline (group B), 100 ug of pVAX1 (group C), 100 ug of pVAX1-*HA2-fimA* (group D), 100 ug of pVAX1-*HA2-fimA/IL-15* (group E), and 100 ug of pVAX1-*HA2-fimA*+CpG-ODN 1826 (30 μg) (group F), respectively. Scale bars represent 20 μm. Magnification, 200×

To evaluate the safety of periodontitis gene vaccines, the major organs, including heart, liver, spleen, lung, and kidney, were collected from SD rats in the groups A–F at week 8. HE staining results showed that no significant abnormities were found in all organs of SD rats treated with the periodontitis gene vaccines with or without adjuvant (Fig. 8).

## Discussion

Periodontitis is one of the most common inflammatory diseases produced by a dysbiotic subgingival microbiota, including *P. gingivalis*, *Tannerella forsythia*, and *Treponema denticola* [6, 34]. Accumulating evidence supports the “keystone-pathogen hypothesis” in which *P. gingivalis* is identified as a keystone pathogen among several bacteria present in this dysbiotic subgingival microbiota, and its colonization triggers dysbiosis and alteration of host immune responses, and other bacteria orchestrate inflammatory disease leading to bone loss [6, 34–36]. Because there are no obvious symptoms in the early stages, patients often already have deep periodontal pocket formation and symptoms of bone resorption by the time they seek medical attention, which can eventually lead to loosening of the teeth [37]. Vaccines are considered to be one of the most economical and effective means to prevent and treat diseases because they can effectively stimulate a protective immune response in the host. In recent years, there is increasing evidence that genetic vaccines, as a new type of vaccines after attenuated and inactivated vaccines, are effective in the prevention and treatment of periodontitis disease [38–42].

We previously developed a gene vaccine (pVAX1-*HA2-fimA*) against *P. gingivalis* to prevent and treat periodontitis [15–18]. However, there are still problems of poor immunogenicity and immunoreactivity of pVAX1-*HA2-fimA*. The emergence of immune adjuvants has solved these problems well. Toll-Like Receptors (TLRs) are a significant class of pattern recognition receptor (PRR), and because of their innate immunostimulatory properties, they have been used as candidate adjuvants for research in vaccines, particularly that which can be modified by genetic engineering or chemically synthesized, like Poly(I:C) (TLR3) and CpG DNA (TLR9) [43, 44]. CpG-ODN is a favorable vaccine adjuvant that mimics the immunostimulatory activity of bacterial DNA and TLR9 signaling and is not immunogenic itself and does not induce autoimmune responses [45]. In this study, CpG-ODN 1826 was used as an adjuvant to study the influence of the immunogenicity of the periodontitis gene vaccine (pVAX1-*HA2-fimA*).

It has been shown that pathogen-specific secretory IgA (SIgA) antibody (Ab) plays a critical role in the protection and homeostatic regulation of the mucosal epithelia [46]. In this study, the levels of FimA-specific and HA2-specific secretory IgA antibodies in the saliva of rats in group I-VI were measured from week 0 to 10, respectively, and the results showed that the SIgA antibody levels in the pVAX1-*HA2-fimA*+CpG-ODN 1826 (30 μg) group were consistently higher than those in the other groups. Therefore, 30 μg could be the optimal immunization dose for CpG-ODN 1826 and used in subsequent experiments (Fig. 3a, b).

Several researches have demonstrated that CpG-ODN had better effects compared to other adjuvants in enhancing the organism's response to antigens, especially for less immunogenic antigens and when the dose of the immunogen was not sufficient to elicit an effective immune response [20, 47, 48]. In this study, we evaluated the specific immune responses and protective efficacy in SD rats given a periodontitis gene vaccine (pVAX1-*HA2-fimA*) plus CpG-ODN 1826 (30 µg) adjuvant to prevent oral infection by *P. gingivalis* and also compared the effect of CpG-ODN 1826 with that of the previously used IL-15 adjuvant. The levels of FimA-specific and HA2-specific secretory IgA antibodies in the saliva of rats in the group A–F were measured from weeks 1 to 8, respectively and the results showed that the levels of SIgA antibody were consistently higher in the pVAX1-*HA2-fimA*+CpG-ODN 1826 (30 µg) group than in the other groups during weeks 1–8 (*P* < 0.05, except week 1 or 2) (Fig. 4a, b). Our results were consistent with these studies, showing the effect of CpG-ODN 1826 as an adjuvant for immunization against periodontitis.

Alveolar bone loss is a prominent feature of periodontitis and is usually used as a marker of periodontitis. Tumor necrosis factor (TNF)-α and interleukin-1β (IL-1β) are inflammatory cytokines involved in periodontitis, and they can affect and amplify the inflammatory response, resulting in tissue destruction and bone loss [49]. COX-2 acts as a mediator in the inflammatory pathways and strongly activates the inflammatory response by releasing pro-inflammatory cytokines like TNF-α and IL-1β [50]. It has been demonstrated that COX inhibitors could significantly reduce alveolar bone loss in periodontitis rats [51, 52]. RANKL, a polypeptide of amino acids, binds to RANK on the cellular membranes of osteoclast precursors. It induces the conversion of osteoclast precursors into mature osteoclasts and promotes their activity, playing an important role in periodontal bone resorption [53]. Kim et al*.* suggested the innate immune response promoted osteoclastogenic activity by activating RANKL via TLR pathways [54]. It has been reported that RANKL-associated periodontal bone loss involved an increase of RANKL-dependent B and T cell response with *P. gingivalis* infection [55]. In summary, COX-2 and RANKL were susceptible markers of inflammation and periodontal tissue destruction in periodontitis. In this study, we evaluated the specific immune responses and protective efficacy in SD rats given a periodontitis gene vaccine (pVAX1-*HA2-fimA*) plus CpG-ODN 1826 adjuvant by measuring alveolar bone loss and immune-inflammatory modulation. A stereomicroscope observation showed that pVAX1-*HA2-fimA*+CpG-ODN 1826 (30 µg) significantly reduced alveolar bone loss in ligated maxillary molars in group F compared with groups B–E (*P* < 0.05) (Fig. 5g). The result of immunohistochemical staining showed that the levels of COX-2 and RANKL were significantly lower in the periodontal tissue of the maxillary second molars of SD rats in group F compared with groups B–E (*P* < 0.05) (Figs. 6g, 7g). In conclusion, treatment with pVAX1-*HA2-fimA*+CpG-ODN 1826 (30 µg) compared to pVAX1-*HA2-fimA* alone or pVAX1-*HA2-fimA* plus IL15 as adjuvant proved its potential in reducing the expression of COX-2 and RANKL in bone, thereby inhibiting inflammation and reducing osteoclast activation and differentiation, thus decreasing bone loss.

Safety is always the top concern in developing a vaccine. As shown in Fig. 8, no significant abnormities were found in all organs of SD rats at week 8 treated with the periodontitis gene vaccines with or without adjuvant. These results were consistent with the previous safety study on pVAX1-*HA2-fimA* and pVAX1-*HA2-fimA/IL-15* [17], indicating that pVAX1-*HA2-fimA* with or without CpG-ODN 1826 was not toxic in the short term for in vivo use.

## Limitations

The concentrations of CpG-ODN 1826 (10, 30, 50 µg) and the number of samples (7 per group) in this study were established with reference to literatures, such as 20 µg of CpG 1826 for 20 mice per group [56] and 50 µg of CpG 1826 for 6 BALB/c mice per group [57]. In general, the precision of the results increased with increasing concentration gradients as well as the number of samples. In this study, there were limitations in terms of concentration gradient as well as sample size.

## Conclusion

In conclusion, the present investigation demonstrated that CpG-ODN 1826 (30 µg) could be used as an effective and safe mucosal adjuvant for periodontitis gene vaccine (pVAX1*-HA2-fimA*) since it could elicit mucosal SIgA responses and modulate COX-2 and RANKL production, thereby inhibiting inflammation and decreasing bone loss.

## Data Availability

Data used and/or analysed during the current study are available from the corresponding author on reasonable request.

## Abbreviations

CpG-ODN: CpG oligodeoxynucleotide
*P. gingivalis*: *Porphyromonas gingivalis*
COX-2: Cyclooxygenase 2
RANKL: Receptor activator of nuclear factor (NF)-B ligand
HA2: Hemagglutinin-2
FimA: Fimbrillin
LPS: Lipopolysaccharide
TLR9: Toll-like receptor 9
SD: Sprague Dawley
TNF: Tumor necrosis factor
IL: Interleukin
PRR: Pattern recognition receptor
HE staining: Hematoxylin and eosin staining
